# Employing T-Cell Memory to Effectively Target SARS-CoV-2

**DOI:** 10.3390/pathogens12020301

**Published:** 2023-02-11

**Authors:** Zaw Htet Tun, Nang Thinn Thinn Htike, Chaw Kyi-Tha-Thu, Wing-Hin Lee

**Affiliations:** 1 Faculty of Medicine, Royal College of Medicine Perak, Universiti Kuala Lumpur (UniKL RCMP), Ipoh 30450, Perak, Malaysia; 2 Department of Immunology, Graduate School of Medicine, International University of Health and Welfare, Narita 286-8686, Japan; 3 Faculty of Pharmacy and Health Sciences, Royal College of Medicine Perak, Universiti Kuala Lumpur (UniKL RCMP), Ipoh 30450, Perak, Malaysia

**Keywords:** T cells, SARS-CoV-2, vaccine, COVID-19, lung TRM

## Abstract

Well-trained T-cell immunity is needed for early viral containment, especially with the help of an ideal vaccine. Although most severe acute respiratory syndrome coronavirus 2 (SARS-CoV-2)-infected convalescent cases have recovered with the generation of virus-specific memory T cells, some cases have encountered T-cell abnormalities. The emergence of several mutant strains has even threatened the effectiveness of the T-cell immunity that was established with the first-generation vaccines. Currently, the development of next-generation vaccines involves trying several approaches to educate T-cell memory to trigger a broad and fast response that targets several viral proteins. As the shaping of T-cell immunity in its fast and efficient form becomes important, this review discusses several interesting vaccine approaches to effectively employ T-cell memory for efficient viral containment. In addition, some essential facts and future possible consequences of using current vaccines are also highlighted.

## 1. Introduction

T-cell immunity is a part of the major defense system of a host against any kind of viral infection. The availability of T cells for memorizing viral peptides from surface and internal proteins makes cell-mediated immunity superior to humoral immunity, where the neutralizing antibodies only recognize the surface proteins of the virus. The two main essential components of T-cell-mediated immunity are CD4^+^ and CD8^+^ T lymphocytes. The purpose of the response of CD4^+^ T cells toward viral infections is to recognize the viral epitope presented on class II major histocompatibility complex (MHC II) molecules of the antigen-presenting cells (APCs) that provide help to other immune cells, thereby yielding an effective cell-mediated immunity. CD8^+^ T cells, in contrast, recognize the viral epitope presented on MHC class I molecules and perform dual functions, including a non-cytolytic effector function that uses their cytokines for the suppression of viral replication, as well as a function to induce apoptosis of the virus-infected target cells by releasing perforins and granzymes [1,2,3]. Since the first wave of the coronavirus disease 2019 (COVID-19), the essential need for T-cell immunity for the speedy viral clearance and suppression of the severity of the disease was clearly identified [4,5]. On the other hand, it was also necessary to pay attention to the virus-induced T-cell defects seen in some severe cases [6,7,8]. However, T-cell abnormalities such as exhaustion and the T-helper 17 cells (Th17)-skewed response have rarely been reported since the first-generation COVID-19 vaccines reached the market and mass vaccination was conducted across the globe. This points out the importance of properly educating the T-cell immunity early before the virus has time to prepare for its hostile establishment. Several vaccines have been developed and have successfully controlled the virus to some extent, but the frequent emergence of mutant strains is still occurring. A strain such as Delta is deadly, whereas Omicron is super infectious with high-speed spread [9,10,11,12]. These mutants can evade the neutralizing effect of a host’s antibodies [13,14,15]; therefore, there is a need to investigate approaches to boost cell-mediated immunity. There is not a long-term promise with the current commonly used vaccines, such as spike (S)-protein-targeted vaccines and inactivated vaccines, to control future mutant strains. If there is an emergence of a virulent strain which is able to set up in respiratory tracts with a high viral load, which could possibly cause T-cell defects within a short period of time, or which could escape from T-cell immunity, there will potentially be another occurring epidemic or pandemic that is accompanied by a high fatality rate. Therefore, a deeper understanding of T-cell immunity toward SARS-CoV-2 is needed. At the same time, better vaccine approaches should be explored to generate the broad-memory T-cell pool, which could be utilized to recognize several common SARS-CoV-2 epitopes that are shared among global communities. In addition, the strategic location of tissue-resident memory T cells (TRM) in the airway also may later play an essential part in finding and implementing better vaccine approaches. Several second-generation vaccines are on their way to fill these holes and gaps based on the experiences of first-generation vaccines. One main aim of these vaccines is how to educate the cell-mediated immunity in the best way to face any mutant strain of SARS-CoV-2. By using T-cell memory with help from an ideal vaccine and vaccination strategy as an advantage, without a doubt, the pandemic spread of SARS-CoV-2 could be stopped or suppressed. Since this adaptive arm of the immune system plays a key role in long-term viral control, we review some essential facts regarding T-cell immunity after exposure to SARS-CoV-2, such as abnormalities including T-cell exhaustion and the Th1/Th2/Th17 imbalance response, as well as the profile of memory T cells seen in COVID-19 cases. In addition, we discuss some essential facts and possible consequences which need to be taken into account when employing T-cell memory to effectively target SARS-CoV-2 through various vaccine approaches.

## 2. Viral-Induced T-Cell Abnormalities in COVID-19

### 2.1. T-Cell Exhaustion

The functional impairment of T lymphocytes in the setting of continuous exposure to an antigen is termed T-cell exhaustion [16,17]. T-cell exhaustion has been reported in relevant chronic persistent infections such as human immunodeficiency virus (HIV), hepatitis B virus (HBV), and hepatitis C virus (HCV) [18]. An essential characteristic of exhausted T cells includes the lack of ability to produce functional cytokines with the sustained expression of multiple inhibitory immune checkpoint receptors (IRs) such as PD-1, TIM-3, cytotoxic T lymphocyte antigen-4 (CTLA-4), lymphocyte activation gene-3 (LAG-3), T-cell immunoglobulin, and ITIM domain (TIGIT) [19]. Severe SARS-CoV-2-infected cases have been reported to have a high frequency of CD-8^+^ T cells, which express multiple IRs such as PD-1, CTLA-4, and TIGIT [6]. The individual expression of PD-1 in some COVID-19 cases [20] seems to be a reflection of activation instead of exhaustion, but the expression of several IRs indicates the possibility of T-cell exhaustion. Moreover, inefficient functionality of the overall CD8^+^ T-cell response, characterized by the low percentage of interferon-gamma (IFNγ^+^) CD8^+^ T cells, CD107a^+^ CD8^+^ T cells, IL-2^+^ CD8^+ ^T cells, and granzyme B^+^ CD8^+ ^T cells, was also detected in COVID-19 cases [21]. In addition to the CD8^+^ T-cell defect, CD4^+^ T cells in COVID-19 cases also express the exhausted markers [22], and a high frequency of non-functional CD4^+^ T cells that are deficient at producing IFNγ, IL-2, and TNFα has been demonstrated in severe COVID-19 cases [6]. One study group suggested that the high viral load is a main trigger factor of T-cell exhaustion by demonstrating the presence of CD8^+^ TIM-3^+ ^CD39^+^ T cells in critically ill COVID-19 cases [23]. Based on previous experiences with human immunodeficiency virus (HIV) and hepatitis C virus (HCV) cases, the expression of CD39 was directly linked with the viral load, and high CD39 expression was found in the terminally exhausted phenotype of CD8^+^ T cells [24]. Hence, although the T-cell exhaustion seen in these chronic viral infections is due to persistent antigenic exposures, T-cell exhaustion in an acute viral infection of SARS-CoV-2 is most likely because of the exposure of T cells to the high viral load within a short time frame. A promising strategy to resolve T-cell exhaustion in SARS-CoV-2 cases may be the usage of immune checkpoint inhibitors (ICIs), such as anti-PD1 and anti-CTLA4, and antiviral drugs in the early stage or as soon as the viral antigen has been detected, but especially before the establishment of an extensive viral replication. One ex vivo study demonstrated the ability of a PD-1 blockade to restore the functionality of exhausted T cells from the convalescent COVID-19 cases by challenging them with the SARS-CoV-2 peptides [25]. Essentially, the potential risk of cytokine release syndrome (CRS) from the usage of ICIs [26] needs to be considered, as it could potentiate the COVID-19 cases with pneumonitis into full-blown acute respiratory distress syndrome (ARDS). Hopefully, future clinical trials will discover the best timings and strategies for using ICIs in accordance with the SARS-CoV-2 viral pathogenesis to impede T-cell exhaustion in COVID-19.

### 2.2. Th1, Th2, and Th17 Imbalance

Once primed with the processed antigenic peptide of the exposed infection, T cells can differentiate into several effector phenotypes such as Th1, Th2, and Th17 [27]. As COVID-19 is caused by a viral infection, the Th1 response, together with the cytotoxic T-cell (CTC) response, is a necessity for viral containment in the acute phase of COVID-19. However, the Th1-driven response is suppressed in critically ill patients with acute COVID-19 [7]. The viral suppression of IFN-1 [28] and virus-induced high-level IL-6 and IL-10 are the major drivers of Th1 suppression [29,30], which could possibly lead to skewing toward the Th2 and Th17 phenotype-dominated response. Evidence such as the high tissue expression of IL-4 in lung biopsies found in deceased COVID-19 cases, in addition to the discovery of eosinophilia, degranulated eosinophils, and basophilia in peripheral blood smears of severe cases, indicates the positive link between the Th2-driven response and the severity of the cases [8,31]. However, a pre-existing strong Th2-driven response seems to act as an alleviating factor of COVID-19, as a smaller chance of hospitalization was recognized among SARS-CoV-2-positive asthmatic cases [32,33]. Therefore, as a hypothesis, the Th2-driven response seen in severe COVID-19 cases may have occurred as a repair mechanism for diffuse alveolar damage rather than acting as an inducing factor of acute respiratory distress syndrome (ARDS). In addition to the Th1/Th2 imbalance, skewing toward the Th17 phenotype-driven response is also implicated in acute COVID-19 cases. In fact, the Th17-skewed effect is the most likely the inducing factor of high severity, especially for the lung pathology. Findings in severe cases such as those with high levels of Th17 cells and high neutrophil counts in peripheral blood, as well as high neutrophil counts in bronchoalveolar lavage fluid, reveal the fact that a Th17-dominant response alongside the recruitment of other inflammatory cells by IL-17 [34,35,36] could be the pivotal trigger factor for diffuse alveolar cell damage, which is accompanied by hyaline membrane formation, stiff lungs, and organizing pneumonia.

## 3. Memory T Cells in COVID-19 Cases

Although some COVID-19 cases have been complicated by T-cell abnormalities such as T-cell exhaustion, the Th17-skewed effect, and lymphopenia, convalescent cases recovered with the efficient generation of memory T cells [37]. From the past experiences of SARS-CoV-1 infections, it is known that memory T lymphocytes can be seen for several years after viral exposure and that these cells were still able to control an incoming SARS-CoV-1 infection while the antibody response faded away several months after viral entry [38,39]. This evidence highlights the essential need for memory T-cell generation for the long-term containment of SARS-CoV-2 in the current pandemic. In a majority of SARS-CoV-2 convalescent cases, memory T cells that respond to several viral proteins can be found in their peripheral blood following a few weeks of viral exposure [40]. The major targeted viral protein of both CD4^+^and CD8^+^ memory T lymphocytes is the S protein, but the memory T-cell recognition of SARS-CoV-2 is not limited to the S protein, as it can also yield a broad respond to the membrane glycoprotein (M) protein, nucleocapsid (N) protein, and several non-structural proteins [37,38,40].

Mild cases are assumed to have effective memory T-cell responses based on the previous reports of the robust virus-specific T-cell responses found in asymptomatic cases, and there is an inverse correlation between the severity of SARS-CoV-2 and the frequency of virus-specific T cells [41,42]. In addition, SARS-CoV-2-specific memory T cells can also effectively target and suppress the SARS-CoV-2 mutant strains such as B 1.1.7, B.1.351, and B.1.1.248 [43,44]. In fact, memory T cells from most convalescent cases can also still respond to the most threatening Omicron variant [45]. Furthermore, current studies have also proved the endurance of the SARS-CoV-2-targeted memory T-cell response, which can be present for up to 10 months [46,47,48]. A recent finding on the reduction in both virus- specific memory CD4^+^ and CD8^+^ T lymphocytes within the initial 5 months [49] has caused alarm regarding the risk of the fast decay of these memory T cells, but it does not necessarily reject the possibility of their persistence for several years. In fact, several studies have highlighted the presence of stem cell-like memory T cells (TSCMs) in convalescent COVID-19 cases [20,41,46]. One group detected a considerable proportion of CCR7^+^ CD45RA^+^ CD95^+^ stem cell-like memory T cells (TSCMs) among SARS-CoV-2-specific memory CD4^+^ and CD8^+^ T cells from the peripheral blood of convalescent cases, and these TSCMs were shown to have the capacity for self-renewal, be polyfunctional, and be able to differentiate into diverse effector and memory T-cell subsets [46]. In other words, these cells can continually replenish the effector memory T-cell pool (Tem) and the terminally differentiated effector T cells (Teff) inside the circulation. Moreover, they can be a source for maintaining the lung’s tissue-resident memory T cells (TRM) [50] which are the most important guards for immediate viral containment. As the delay or poor response of T-cell immunity is one of the possible causes of severe lung inflammation and a cytokine storm in severe COVID-19 cases, having SARS-CoV-2-specific TSCMs that could rapidly recharge the Tem cells, Teff cells, and especially the lung TRM cells is ideal for inducing the rapid response of efficient cell-mediated immunity. From these TSCMs and the Tem pool of virus-exposed individuals, future studies need to find other precise and specific ways, for example, an ideal vaccine approach, to rapidly refill effector cells in peripheral blood, as well as to recruit TRM cells to the viral burden sites such as the respiratory tract, lungs, and gut for early viral suppression and containment.

## 4. Employing T-Cell Memory to Effectively Target SARS-CoV-2

Several vaccine approaches have been tried to shape the T-cell memory into its most effective form. The most widely used first-generation vaccines include mRNA vaccines (mRNA 1273 and BNT162b2 mRNA), vector vaccines (Sputnik and AZD1222), and inactivated vaccines (BBIBP-CorV and CoronaVac). Most of these vaccines can induce the Th1-skewed response that is accompanied by the CD8^+^ T-cell response, which is a favorable response for effective viral containment [51,52,53,54,55,56,57]. The functionality of T cells has been confirmed by demonstrating their ability to produce interleukin-2 (IL-2), IFNγ, and TNFα [51,52,58,59]. As the first-generation mRNA vaccines and vector vaccines do not encode the M protein and N protein, these vaccines can only induce the S-protein-specific T-cell response rather than a broad response to other structural proteins. However, in individuals who received the inactivated vaccines, T cells specific to M and N proteins can be seen together with the S-protein-specific T cells [57,59]. This kind of broad response, as opposed to the isolated spike-specific T-cell response, is comparable to the virus-specific T-cell response found in convalescent cases [37,40], and it generally is assumed to be important for the containment of future mutant strains. However, the magnitude of the T-cell response to inactivated vaccines is quite low, although it can still manage SARS-CoV-2 [57]. In contrast, mRNA vaccines and vector vaccines can induce a potent T-cell response that specifically targets the S protein. In addition, an efficient germinal center (GC) response with the active involvement of follicular helper T cells (Tfh), B lymphocytes, and plasmablasts was well-recognized in response to the mRNA vaccines [60,61]. Indeed, this modified mRNA-lipid nanoparticle (LNP) formulation platform not only enhances the host-cell uptake of the mRNA, but it also helps with the dissemination of mRNA to several lymph nodes, which further facilitates the possibility of direct antigen expression by the lymph node (LN)-resident antigen-presenting cells and B cells to the naive T cells for efficient Teff and Tfh responses [62,63,64,65,66]. The vector vaccines can also induce the Teff cell response, but Tfh response has not been well-reported.

Although most first-generation COVID-19 vaccines have targeted the S protein as the major immunogen, some studies have highlighted the possibility of implementing a better option to induce efficient broad T-cell memory by adding other viral proteins, especially the N protein in addition to the S protein [4,67,68,69]. It is true that the spike has been well-recognized as the key protein because of its essential role in mediating viral entry into the host cells as well as its ability to induce potent neutralizing antibodies [67,70,71]. Nevertheless, the frequent emergence of SARS-CoV-2 spike mutant strains has highlighted the need to find other approaches for effective long-term viral containment. Pfizer and Moderna have tried with Omicron-specific vaccines, but their effectiveness against the original Wuhan strain and future mutant strains is doubtful. Thus, it is important to educate the T-cell memory to be able to broadly recognize other viral proteins. Adding an N protein to the S protein could help build a broad-based T-cell memory that can target several viral epitopes. Essentially, the N protein has more conserved regions compared to the S protein, and it also has been suggested as one of the potential targets for vaccine development [72,73]. Nevertheless, the proportional percentage of N-protein-specific CD4^+^ T cells and CD8^+^ T cells from the total virus-specific T-cell response is quite low, and the T-cell response is widespread across several targets of SARS-CoV-2 [38,40,74,75]. In other words, the N protein targeting T-cell response alone will not be sufficient to manage SARS-CoV-2. Therefore, some second-generation vaccines are trying to introduce T-cell immunity with the N protein as well as other structural proteins such as the M protein and open reading frames (ORFs), together with the S protein [76,77,78]. One promising second-generation vaccine is the Gritstone COVID-19 vaccine, which uses self-amplifying mRNA (SAM) that encodes highly conserved non-spike T-cell epitopes (TCE) in addition to the S protein. The first cohort of Vaxzevria (formerly the Oxford-AstraZeneca, UK)-vaccinated adults (>65 years old) that received the Gritstone vaccine as a booster showed the promising result of a broad T-cell response toward the highly conserved non-spike T cell epitopes (TCE) of the N protein, M protein, and ORF3a; the TCE-targeted T-cell proportions were demonstrated by ELISpot assay as 36% (N protein), 22% (M protein), and 42% (ORF3a) [76]. Adding the M protein as an additional target in this approach can trigger a broader T-cell response, which could possibly be cross-reactive across several SARS-CoV-2 mutant strains and other coronaviruses, although the M protein itself is not highly immunogenic [79]. Another interesting approach can be seen in the “dual-antigen T-cell vaccine” from ImmunityBio, which is constructed with S and N proteins by using the human adenovirus type 5 vector (Ad5). For powerful T-cell stimulation, “Enhanced T-cell Stimulation Domain” (N-ETSD) is added along with the N protein for the better expression of the viral antigen on MHC molecules, as ETSD can help navigate the N protein toward the endo/lysosomal compartment. In addition, a signal sequence that facilitates the better cell-surface expression of spike is combined with the full-length spike protein in this vaccine to induce robust humoral and cell-mediated immunity [77,78,80]; moreover, the E1, E2b, and E3 genes of the hAd5 vector in this vaccine are removed to prevent anti-vector immunity in the recipients. A single booster of the dual-antigen vaccine in previously infected cases was shown to recall the memory CD8^+^ T lymphocytes, as well as the Th1-dominant S- and N-ETSD-targeted T lymphocytes [77,78,81].

Although targeting several proteins is a general way to prevent viral escape, one important thing to consider is that some epitopes are immunogenic, whereas others may be immunopathogenic due to the individual variation in human leucocyte antigen (HLA) types. The HLA-A* 02:01-restricted epitope linked with the suboptimal T-cell response [82], a lack of memory T-cell recognition toward the P272L epitope presenting in HLA A*02^+ ^cases [83], and the dysfunctional CD8^+^ T-cell response to the HLA-A*01:01-restricted epitope [84] are some examples of the implications of HLA in a poor T-cell response. If these epitopes act as immunodominant epitopes that are able to draw on most of the TCR repertoire of an infected host, it is possible that the overall T-cell immunity will become inefficient. In future vaccine design, epitopes such as these should be removed to maintain the target of memory T cells on other immunogenic epitopes. At the same time, some epitopes that are highly immunogenic in the yielding of the functional memory T cells can also become immunodominant [85]. Therefore, T cells should be primed early with the peptides that represent immunogenic epitopes. In fact, an analysis of the epitope specificity of T cells in convalescent cases does not completely represent the T-cell immunity in the acute phase. Longitudinal studies should be carried out to explore the timing of the expressed epitope and its impact on T-cell immunity, especially those immunodominant epitopes that appear early in the course of infection. In addition, understanding the immunological hierarchy and specific role of these conserved viral epitopes of the targeted HLA types could help establish a more focused T-cell memory, which could prevent frequent viral escape. It is unreasonable and tiring to find all HLA-restricted epitopes from all HLA types. Nevertheless, at least the immunogenic epitopes that are conserved among highly prevalent HLA supertypes [86,87] should be uncovered to implement a globally effective vaccine approach for SARS-CoV-2. If possible, it is better to find some epitopes that can be presented on both HLA class I and class II, as they can trigger a more focused and harmonized response of CD4^+^ T lymphocytes and CD8^+^ T lymphocytes that target the same specific site. In this way, the non-specific, cross-reactive CD4^+^ T-cell response that recognizes long peptides on an MHC class II molecule will also be limited to some extent. Using this approach of targeting conserved epitopes also highlights the possibility of finding a pan-coronavirus vaccine to target shared epitopes across all coronaviruses. However, there are some limitations. Firstly, the specificity of the antigen-specific private TCR repertoire to other coronaviruses is hard to estimate. When the host is exposed to a specific viral epitope, the TCR repertoire specific to that epitope establishes the memory toward the common public complementarity-determining region 3 (CDR3) motif. However, the specific sequence and length of CDR3 in this motif varies between each individual, which is known as private TCR sequences [88]. Because of this, the contribution of the antigen-specific TCR repertoire to all coronaviruses will be hard to estimate in terms of specificity. In addition, the next coronavirus may carry similar epitopes, but if there are other more immunodominant epitopes, the previously primed T-cell memory will not be able to target these new immunodominant epitopes.

**Figure 1:**
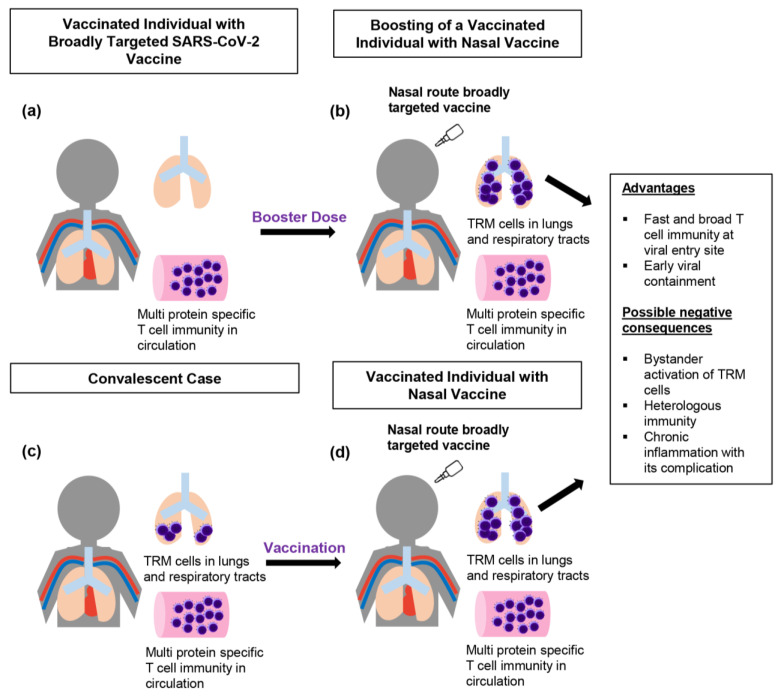
A schematic diagram of vaccine strategy for an efficient T-cell immunity and its possible long-term consequences. (**a**) T cells should be primed with an ideal vaccine that includes several viral proteins such as structural and non-structural proteins to be able to broadly target SARS-CoV-2, a similar response which can be seen in convalescent COVID-19 cases. In the future, vaccine strategies should be improved by targeting specific immunodominant and immunogenic epitopes. (**b**) Nasal vaccine as a booster dose is a promising strategy to induce TRM cell response at the viral entry sites such as the respiratory tract and lungs in addition to the systemic T-cell immunity. (**c**) Convalescent cases may have TRM cells in the lungs, but the cell number will decrease over time. (**d**) The frequency of lung TRM levels in convalescent cases can be restored by using the nasal vaccine approach (**b**,**d**) Vaccination by nasal route is a promising strategy to recruit virus-specific T cells to the respiratory tract. A swift CD8^+^ TRM response by intranasal vaccine as a booster can effectively control the virus at the respiratory tracts and lungs. However, there are some potential negative consequences of the intranasal approach such as non-specific bystander activation of TRM cells, heterologous immunity to similar epitopes, and chronic inflammation with its long-term complications such as metaplasia and dysplasia.

While shaping the T-cell memory to target specific epitopes of the structural proteins and non-structural proteins, it is also essential to develop the memory T-cell response at the strategic site of viral entry. Among the alternative routes of vaccination such as oral, nasal, and skin nano-patches tried by the next-generation COVID-19 vaccines [89,90,91,92], the nasal route is an attractive option as this approach can directly target the mucosal T-cell immunity of the upper respiratory tract, lower respiratory tract, and lungs (Figure 1), which are the boarding sites of SARS-CoV-2. By establishing strong mucosal immunity in the airway, theoretically, the viral load could be suppressed, and it would be less likely that the high viral load would move down to the lungs. Findings on, for example, the intense viral shedding from SARS-CoV-2-infected airway cells [93] point out the need to build up strong local immunity in these areas. It is true that T-cell immunity can be induced there and maintained for several months. In convalescent COVID-19 cases, virus-specific TRM cells can be found in lung parenchyma for up to 10 months after SARS-CoV-2 exposure [94], but the frequency of these cells will decrease over time. Therefore, it is also essential to refill TRM in the lungs by using the nasal approach (Figure 1). Several preclinical studies reported the ability of nasal SARS-CoV-2 vaccines to trigger a T-cell response in the airway and lungs [89,90,95,96]. Interestingly, functionally active CD103^+^ CD69^+^ CD8^+^ memory T cells in addition to IFNγ and granzyme B-producing T cells are successfully induced in the respiratory tracts by an S-protein-based nasal vaccine delivered with a ChAd vector, whereas an intramuscular vaccine failed to yield the same result [95]. Intramuscular injections can definitely yield systemic immunity, but they are not the best solution to provide strong local mucosal immunity in the upper and lower airways. Most of the previous studies have judged vaccine-induced T-cell immunity by using the peripheral blood, but the fact is that the response seen in the circulation does not paint a comprehensive picture of the whole-body T-cell immunity, especially with regard to the profile of tissue-resident memory TRM cells. The memory T-cell pool in the circulation could replenish the lung TRM cells to some extent, but introducing viral epitopes via the nasal route is more reasonable to directly recruit TRM cells to the lungs. The better results of the nasal vaccines were later revealed based on their effectiveness as a booster after the hosts were primed with the intramuscular (IM) vaccine [89,90]. A heterologous prime-boost approach using intranasal spike boosting can successfully elicit the lung tissue-resident TRM cells, including both CD4^+^ TRM and CD8^+ ^TRM cells, together with class-switched virus-specific antibody-secreting B cells that express immunoglobulin A (IgA) and immunoglobulin G (IgG) [89]. Thus, the nasal route is an essential requirement for recruiting memory T cells, especially TRM cells, to the airways and lungs, which could elicit an efficient T-cell response within a short time frame upon exposure to a new SARS-CoV-2 infection; this could not only decrease the viral load, but also reduce transmissibility by suppressing viral shedding. Later, a study of an intranasal vaccine called the trivalent ChAd vector vaccine that carries not just the spike, but also includes the N protein and truncated non-structural protein 12 (nsp12) of SARS-CoV-2 showed its ability to control some variants of concern such as B.1.1.7 and B.1.351 [90], demonstrating it to be a promising potential strategy to be used for future mutant strains since this trivalent nasal vaccine induces functionally active multi-epitope-specific CD8^+ ^T cells and an effective Th1-skewed response in the airway. There are several nasal vaccines that are currently being tested in clinical trials [97,98,99,100,101]. As a first sign of success, an encouraging result of strong T-cell immunity that could recognize >99.2% of the Omicron (BA.2) peptide pool was reported for the live-attenuated nasal vaccine from Codagenix [100]. Hopefully, some of these nasal vaccines will be authorized soon and released to the market, as these vaccines are not just effective, but they have several advantages such as being easy and flexible to produce, not requiring medical professionals for distribution, and being able to be stored inside the fridge for long durations.

Although we will hopefully achieve better outcomes by using nasal vaccines, some possible pitfalls and negative consequences have to be taken into account. Firstly, a high number of TRM cells in the lungs does not necessarily lead to exclusive protection from the viral infection. For example, TRM cells in obese people may be unable to perform their effector function, as obesity can form a unique T-cell metabolic impairment that can lead to a dysfunctional T-cell response [102]. Similarly, chronic hyperglycemia as a causal factor of memory T-cell dysfunction was demonstrated in type 2 diabetic mice models [103]. In addition, the impaired ability to perform cytotoxic functions alongside a significantly reduced production of granzymeA (GzmA) and perforin in a subpopulation of effector memory CD8^+^ T cells was also reported in aging COVID-19 patients [104]. These go beyond the nasal vaccine’s level of ability to induce lung TRM responses. In addition, although the induction of viral epitopes with the frequent usage of the nasal vaccine may be important for the recruitment of TRM cells to the lungs, these cells need a specific site to stay inside in the harsh environment of the respiratory tract and ill-defined interstitium of the lungs. According to a study using murine models, specific niches can be formed at the peribronchiolar foci while repairing the tissue after the virus-induced damage [105,106]. These niches, called repair-associated memory depots (RAMDs), are the sites that can host CD8^+^ TRM cells. Although the nasal vaccine can strategically recruit the CD8^+^ TRM cells to these RAMDs, most of these RAMDs disappear during tissue regeneration, which could reduce the effectiveness of the nasal approach. On the other hand, the CD4^+^ TRM cells can reside in the inducible bronchus-associated lymphoid tissue (iBALT), which could last for several months [105,106,107,108]. However, the persistent presence of TRM cells inside the lungs can also have potential negative consequences (Figure 1) that can trigger immunopathological changes. For example, in asthmatic people, allergen-specific CD4^+^ TRM cells represent a dominant group residing in iBALT, and the bystander activation of these cells can occur when exposed to the viral infection. One study reported that asthmatic human subjects challenged with respiratory syncytial virus (RSV) could not only virus-specific T-cell immunity, but also the bystander activation of allergen-specific memory Th2 cells, which occurs without TCR ligation [109]. Similarly, one group also demonstrated the bystander activation of lung parenchyma-located virus-specific CD8^+^ TRM cells, but not of the TRM cells from the lungs’ vasculature, in response to the bacteria infection by challenging it intranasally with the bacteria and bacterial product lipopolysaccharide (LPS) [110]. Although bystander activation of virus-specific TRM cells in this study non-specifically alleviated the bacterial infection-induced pneumonia, the ability of the bystander T-cell activation to recruit neutrophils triggered the inflammatory microenvironment inside the lungs. Based on this evidence, environmental allergens, bacterial infections, and bacterial products may possibly cause the bystander activation of nasal vaccine-induced CD8^+^ TRM in the absence of the previously primed viral antigen. Essentially, if these TRM cells are super-active and provide a heterologous immune response to similar epitopes coming from environmental allergens, or if they respond to several environmental antigens via bystander activation, they may initiate the frequent activation of alveolar macrophages and other immune cells, for example, eosinophils and basophils. Consequently, the lungs will have to suffer from the repetitive inflammation, and the chronic inflammatory reaction could be accompanied by fibrosis and tissue damage. Theoretically, if there is silent chronic inflammation for years, it could lead to metaplastic changes, dysplasia, and neoplasia. Notably, these complications such as metaplasia and dysplasia are possibly driven by chronic inflammation due to the frequent usage of nasal vaccines that induce the long-term stay of lung TRM cells, and they will be more pronounced in smokers and people with chronic obstructive pulmonary disease (COPD), as these people already have an unhealthy microenvironment in the respiratory epithelium, which is a fertile place for the development of neoplastic changes upon exposure to inflammation-inducing antigens. The purpose of nasal vaccines is to stop the COVID-19 pandemic, but other negative outcomes and possible pitfalls need to be addressed.

## 5. Conclusions

It is true that T-cell immunity needs to be trained with an ideal vaccine. Without a doubt, if a highly mutant SARS-CoV-2 strain appears in the future, a vaccine that could induce mucosal immunity with a swift and broad T-cell response in the airway and lungs, and that could induce strong systemic immunity could suppress the viral load and halt the pathogenesis. This would result in fewer fatalities and prevent a pandemic. Current vaccine approaches are trying to produce T-cell memory by broadly targeting the several proteins of SARS-CoV-2. Hopefully, future studies may find more conserved regions from viral proteins or a specific peptide pool to be packaged into a vaccine to prime the T cells for a more focused response. On the other hand, a vaccine approach using the nasal route is also a promising method to induce a fast, durable T-cell response. However, the long-term consequences of the presence of potent and fresh TRM cells inside the lungs via the frequent administration of nasal boosters must be investigated carefully. The whole world has witnessed the rapid progress in finding effective vaccines since the early wave of COVID-19. Current vaccine approaches seem mature and are in line with our understanding of the viral pathogenesis and immune response to SARS-CoV-2 and its mutant strains. However, it would be better if an ideal vaccine or vaccine strategy that could induce harmonized humoral and cell-mediated immunity was able to fully stop the SARS-CoV-2 pandemic. While we are waiting for this, as frequent SARS-CoV-2 vaccination becomes somewhat routine, vaccine-induced molecular and immunological changes, especially in T-cell immunity, are essential to analyze in detail.
